# Identification of Aluminum Responsive Genes in Al-Tolerant Soybean Line PI 416937

**DOI:** 10.1155/2010/164862

**Published:** 2010-10-03

**Authors:** Dechassa Duressa, Khairy Soliman, Dongquan Chen

**Affiliations:** ^1^Department of Natural Resources and Environmental Sciences, Alabama A&M University, Normal, AL 35762, USA; ^2^Biostatistics and Bioinformatics Unit, Comprehensive Cancer Center, Division of Preventive Medicine, Department of Medicine, The University of Alabama at Birmingham, Birmingham, AL 35294-3300, USA; ^3^School of Medicine, Clinical and Translational Institute, West Virginia University, HSC-RM-5523, Morgantown, WV 26506-9161, USA

## Abstract

Soybean is one of the most aluminum (Al) sensitive plants. The complex inheritance of Al tolerance trait has so far undermined breeding efforts to develop Al-tolerant soybeans. Discovering the genetic factors underlying the Al tolerance mechanisms would undoubtedly accelerate the pace of such endeavor. As a first step toward this goal, we analyzed the transcriptome profile in roots of Al-tolerant soybean line PI 416937 comparing Al-treated and untreated control plants using DNA microarrays. Many genes involved in transcription activation, stress response, cell metabolism and signaling were differentially expressed. Patterns of gene expression and mechanisms of Al toxicity and tolerance suggest that *Cys2His2* and *ADR6* transcription activators, cell wall modifying enzymes, and phytosulfokines growth factor play role in soybean Al tolerance. Our data provide insights into the molecular mechanisms of soybean Al tolerance and will have practical value in genetic improvement of Al tolerance trait.

## 1. Introduction

Aluminum (Al) toxicity is a major constraint of crop production on acid soils. In view of the fact that 40% of world's arable land is acidic [1, 2], Al toxicity remains a major hurdle for increasing world food, fiber, and fuel production particularly via expansion of cultivation into acid soils. 

Aluminum inflicts a wide range of cellular injuries in plants that ultimately result in reduced root growth, nutrient and water uptake, and productivity [1, 2]. Plants possess some degree of tolerance to Al toxicity that varies among species and genotypes [1, 3–6]. Al tolerance mechanisms include exclusion and internal detoxification. Al exclusion via rhizosphere Al-organic acid anion complex formation is the most widely documented physiological mechanism of Al tolerance in cultivated and wild plants alike [1, 7]. Root-exuded citrate, malate, and oxalate are the key organic acid anions involved in such mechanism. Genes involved in Al-induced root exudation of malate and citrate have been cloned in wheat [8] and sorghum [5], and their variants are being discovered in several plant species. Internal detoxification mechanisms involve the formation of Al complexes with organic acids, acidic polypeptides, and/or proteins and subsequent sequestration of Al in organelles away from sensitive sites in the cell [9, 10]. The genetic components of the internal detoxification pathways are yet to be elucidated.

In soybean, Al tolerance is a complex trait perhaps involving several genes and pathways [11, 12]. Quantitative trait loci (*QTL)* mapping in a population derived from Al tolerant PI 416937 and Al sensitive Young has revealed five DNA markers associated with Al tolerance [11]. Most of the alleles were derived from Al-tolerant PI 416937. Other reported soybean Al tolerance genes include phosphoenolpyruvate carboxylase (*PEPC*), homolog of translationally controlled tumor proteins (*TCTPs*), inosine 5′-monophosphate dehydrogenases (*IMPDHs*) [13], aluminum-induced 3-2 (*Sali3-2*), and aluminum-induced 5-4a (*Sali 4-5a*) [14]. Ermolayev et al. [13] and Ragland and Soliman [14] used gene expression as a tool to identify the above genes but the techniques used in these experiments were not sensitive enough to detect large number of genes that might be expected from the quantitative nature of soybean Al tolerance trait. The objective of this study was to discover putative Al tolerance genes in Al-tolerant soybean line PI 416937 using DNA microarrays—a robust genome—wide transcript profiling technology. Such an approach was recently employed in wheat [15, 16], maize [17], Arabidopsis [18], and *Medicago truncatula* [19, 20] to discern the molecular basis of Al tolerance in the respective species.

## 2. Materials and Methods

### 2.1. Plant Genotype and Growth Conditions

An Al-tolerant soybean plant introduction (PI 416937) highly characterized for Al response [12, 21] was used in this experiment. Seeds were surface sterilized with 20% household bleach (Clorox) in water for 12 min, rinsed with distilled-deionized water several times, and were germinated in deionized water moistened standard germination paper at 25°C in an incubator for 72 h. Seedlings uniform in tap root length were transferred to black-painted pots filled with approximately 4 L of 800 *μ*M CaCl_2_ background solution with 10 *μ*M Al added (treated) or no Al added (control) in a Conviron growth chamber (16/8 h light/ dark cycle with respective temp. of 28°C/20°C, photosynthetic photon density of 100 *μ*mol m^−2^ s^−1^). The pH of the culture solution was adjusted to 4.3 and maintained at that level for the entire duration of the experiment. After 2, 12, 48, or 72 h of Al treatment 1 cm sections of the primary root tips of approximately 15 plants/pot were harvested, immediately flash frozen in liquid nitrogen, and stored at −70°C for RNA extraction. Three independent replicates were used per treatment.

### 2.2. RNA Extraction, Microarray Procedure, and Data Analysis

Total RNA was extracted from 100 mg root tissue samples using Qiagen RNeasy plant RNA isolation kit following the manufacturer's protocol (Qiagen, Inc.). The Affymetrix GeneChip Soybean Genome Array with over 68 000 probe sets, *Glycine max L*. and wild soybean combined, was used for microarray analysis of the soybean genome for Al tolerance. Three chips were used per treatment. Detailed procedures for RNA labeling and array analysis are described in the Manufacturer's GeneChip Expression Technical Manual (Affymetrix). Briefly, the quality of total RNA was determined using the RNA 6000 Nano Chip on Agilent BioAnalyzer 2100 prior to double-stranded cDNA synthesis. Total RNA in the amount of 2 *μ*g was used for double-stranded cDNA generation by linear amplification using oligo dT-T7 primer and reverse transcriptase (RT). Subsequently, biotin-labeled cRNA was synthesized by *in vitro* transcription (IVT) using the ENZO High Yield IVT kit (ENZO). Quality and quantity of cRNA were assessed using the RNA 6000 Nano chip on Agilent BioAnalyzer 2100. Fifteen-microgram cRNA was used for hybridization. Arrays were hybridized overnight at 45°C for 16 h in GeneArray Hybridization Oven 640 (Affymetrix). The next day, arrays were washed and stained in the Fluidics Station 450 (Affymetrix) and scanned by the High Resolution GeneChip Scanner 3000 (Affymetrix).

Gene expression values were determined using theGeneChip Operating Software (GCOS 1.1, Affymetrix). The expression levels were subjected to data query and data mining in Data Mining Tool (DMT). Statistical Analysis of the data was conducted using the software packages ArrayAssist Enterprise together with Pathway Assist (Stratagene/Agilent, Santa Clara, CA). The raw GeneChip files from GeneChip Operating Software (GCOS, Affymetrix, CA) were uploaded, background-subtracted, variance stabilized, and normalized with GC-RMA method [22]. The control group was used as a baseline to calculate the intensity ratio/fold changes of the treatment versus control. The ratio was log_2_-transformed before further statistical analysis. The *P*-values were obtained by an unpaired *t*-test assuming unequal variance. Significantly upregulated and downregulated genes were annotated using protein databases accessed by blastx at National Center for Biotechnology Information (NCBI).

### 2.3. Quantitative Real-Time PCR

Quantitative real-time PCR quantification of transcript levels for representative genes [Gma. 20326: F-5′-tcactccccaccttatcgag-3′, R-5′-tcatgtggtggagtgtggtt-3′; Gma. 6948: F-5′-ttatctccggcgaaaacctc-3′, R-5′-tcgtggtgcagcagtttaag-3′; Gma.12326: F-5′-agccactcaaatggttcagc-3′, R-5′-tctccttgtccttctccttcc-3′; Gma. 24062: F-5′-tgccgaaggatcatctcaac-3′, R-5′-cgagggataatggttgatgg-3′; Gma.26937: F-5′-tacccaaaaggcaggcatac-3′, R-5′-ggccgaggtacaaacacatc-3′; Gma.4156: F-5′-tccaatgctgacaagtgctc-3′, R-5′-tagggacactccgtccaatc-3′; Gma.2577: F-5′-acgcctatgaacgtgaaacc-3′, R-5′-aacatcagcggagagcattc-3′] from microarray experiments was conducted using the Roche Diagnostics light Cycler 480 System with SYBR green detection (Roche Diagnostic, Corp) using beta-tubulin gene (beta-tubulin: R-5′-CCATCAAACCTCAAGGAAGC-3′, F-5′-TGCTGTCCTCTTGGACAATG-3′) as internal control. mRNA was isolated from plants grown under similar experimental conditions as in the microarray experiments. mRNA extraction and quality test was as described above. RNA samples were treated with Applied Biosystems Turbo DNA-free DNase (Ambion, Inc.) to remove DNA contamination. Briefly, 2 *μ*l 10x DNase I buffer and 1 *μ*l rDNase I were added to 20 *μ*l RNA sample, and the mix was incubated at 37°C for 30 minutes in water bath. Subsequently, 2 *μ*l resuspended DNase inactivation reagent was added and the samples mixed well and incubated at room temperature for 3 minutes. Samples were then centrifuged at 10 000 g for 1.5 min (Eppendorf centrifuge 5415 D) in 1.6 ml centrifuge tubes and supernatants transferred to fresh tubes.

cDNA was synthesized from 1 *μ*g DNase-treated RNA samples using the Roche Diagnostics Transcriptor First Strand cDNA Synthesis Kit (Roche Diagnostics, Corp) according to manufacturer's protocol. cDNA concentration and quality was determined using NanoDrop Spectrophotometer brand ND-1000 (NanoDrop Technologies, Inc.). cDNA samples were diluted with nuclease-free water in varying ratios ranging from 1 : 4 to 1 : 10 depending on sample concentration. A total reaction volume of 11 *μ*l comprising 2 *μ*l cDNA sample, 2 *μ*l each of the reverse and forward primers at 0.2 *μ*M concentration, and 5 *μ*l SYBR mix was prepared in 96-well plates (Roche Diagnostics) in two biological and three technical replicates for each gene. A real-time PCR profile of preincubation at 95°C for 5 min, a 45-cycle amplification at 95°C for 10 second, 55°C for 20 second, and 72°C for 20 second, melting at 95°C for 1 min, 65°C for 1 min, and 95°C continuous, and cooling at 40°C for 30 seconds was used to amplify the samples. Negative controls in which cDNA sample was replaced with PCR grade water for each primer pair were included in each run. Sample wells were individually assessed for data quality by evaluating amplification curves and PCR product specificity was verified by melting curve analysis. The expression level of target genes was normalized using in-run beta-tubulin gene as internal control, and transcript concentration ratios were calculated using the ΔΔC_T_-Method [23]. The change in gene expression levels (fold change) was calculated as treatment to control ratio and compared with results from microarray.

## 3. Results and Discussion

### 3.1. Gene Expression in Response to 2-Hour Al Treatment

**Figure 1:**
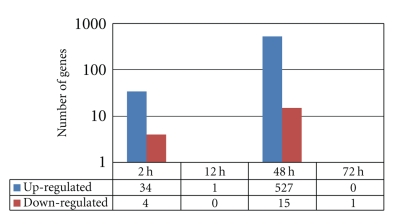
Number of Al upregulated and downregulated genes in soybean genotype PI 416937 in a time-course experiment.

A total of 38 genes were identified as differentially expressed in the 10 *μ*M Al-treated experimental plants compared to no Al added controls at 2 h post Al treatment (Figure 1). Thirty-four of them were upregulated and 4 were downregulated with a fold change ranging from 3.08 to 32.55 (Table 1).

### 3.2. Gene Expression in Response to 12- and 72-Hour Al Treatment

At 12 and 72 h post treatment only one gene each showed significant change in expression in response to Al treatment (Figure 1 and Table 1). 

### 3.3. Gene Expression in Response to 48-Hour Al Treatment

**Figure 2:**
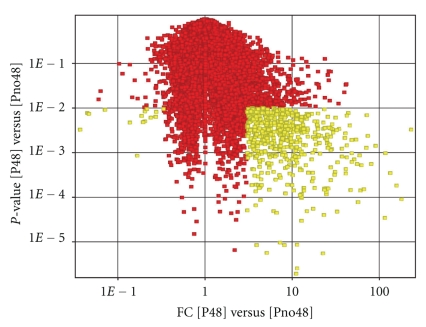
Distribution of *P*-value and fold change for gene expression profile of soybean genotype PI 416937 48 h post treatment. Yellow squares are aluminum regulated genes. Red squares are genes with above back ground expression level. FC, fold change; [P48] versus [Pno48], 48 h treated versus control comparison. *P*-value: the probability that the observed results are obtained by chance not due to Al effect.

The highest number of differentially expressed genes was detected at 48 h post Al treatment (Figures 1 and 2). A total of 542 genes (97.2% upregulated and 2.8% downregulated) were detected. Those exceeding 13-fold changes are presented in Table 2. The marked fold differences observed in the current research are substantially higher in comparison with results obtained by most authors but are comparable to results of [18, 24]. There were two genes in common between the set of genes detected at 2 h and 48 h post treatment (Gma.2577, 7-fold downregulated at 2 h and 8-fold upregulated at 48 h and Gma.26937, 8-fold downregulated at 2 h and 115 upregulated at 48 h). Similar patterns of gene expression were observed in Arabidopsis roots under Al stress with few overlaps between sets of genes detected at 6 h and 48 h post Al treatment [18]. 

The temporal pattern of Al-induced gene expression changes observed in this study diverges from results of other authors. At 12 and 72 h, almost no genes were differentially expressed or detected. The virtually no detection of Al-regulated genes at 12 and 72 h post treatment seems a little odd but it is what is expressed in this soybean genotype at detection thresholds of *P*-value <.01 and 3-fold change in an experiment with 3 replications. Gene expression is species and genotype specific [15–20] making comparison of results across different studies difficult. The most likely explanation for the 72-hour result is that Al toxicity could have already been neutralized by the 72 h, making differential gene expression unnecessary. The lack of transcriptional response at 12 h, however, is a biological puzzle, and it could represent a very unusual temporal transcriptome response of this soybean genotype to Al stress. Among the few reported Al microarray studies, the results of Kumari et al. [18] in Arabidopsis is the closest to ours with regard to the number of genes detected at early and late time points. They detected 127 genes at 6 h post treatment and 733 genes at 48 h post treatment using a threshold of a 2-fold change whereas we detected 38 genes at 2 h and 542 at 48 h using a 3-fold change. 

**Figure 3:**
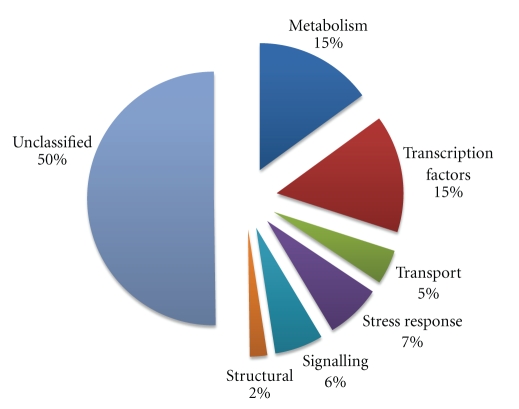
Functional classifications of genes differentially regulated by aluminum in soybean genotype PI 416937.

All of the differentially expressed genes that were functionally annotated by the Genbank nonredundant protein database were grouped into five functional categories based on their putative cellular function. The functional classification showed that stress- and metabolism-related genes constitute the major fractions of Al-regulated genes (Figure 3). 

### 3.4. Quantitative Real-Time PCR Validation of Microarray Expression Levels

**Figure 4:**
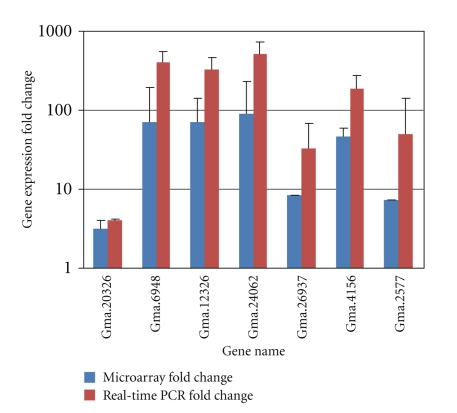
*RT-PCR* gene expression values for representative genes and its correlation with microarray data. Genes were selected to represent the range of expression levels observed in microarray data. Gene expression fold change: ratio of gene expression of Al-treated plants to untreated controls. Data presented are mean ± SE.

The microarray gene expression levels were validated with quantitative real-time PCR for representative genes (Figure 4). In general, the microarray results were in agreement with *qRT-PCR* but in a few cases quantitative *RT-PCR* gave higher levels of expression compared to microarray. Such results are obtained by a number of investigators [16, 20, 25]. Detail discussion of factors contributing to the discrepancy between microarray and *RT- PCR* gene expression levels is covered in [26]. Many authors attribute the phenomenon to the high dynamic range and greater sensitivity of PCR detection. It is worth noting that the gene expression kinetics depicted in Figure 1 shows the efficacy of our experimental design in capturing the full dynamic range of gene expression profiles in the soybean genotype studied. Gene expression peaks at 2 and 48 h suggesting that major savings in microarray experimental expenditure could be realized by limiting sampling to these time points in future experiments. 

### 3.5. Differentially Regulated Genes by Functional Category

#### 3.5.1. Genes Related to Transcription Factors

A number of transcription factors including *bZIP, WRKY, MYB, ADR6,* and *NAc* were highly upregulated in the present study (Tables 1 and 2). Members of these families of transcription factors were previously detected under Al stress in several plant species [16, 18–20, 27]. *Cys2His2*-type zinc finger (*bZIP)* and auxin downregulated (*ADR6*) factors are particularly interesting from Al tolerance perspective. *Cys2His2*-type zinc finger (*bZIP*) protein coregulates molecular response to proton and Al toxicities [28]. It controls the expression of *AlMT1*—a malate transporter protein that acts in Al exclusion mechanism. In this study, *Cys2His2* (Gma.4526, Table 2) was upregulated 51-fold at 48 h post treatment suggesting that malate plays a major role in Al tolerance mechanism of PI 416937 soybean. Earlier physiological study by Silva et al. [29] showed that Al stress increases exudation of both malate and citrate during the first 6 h of exposure to Al in both tolerant and sensitive soybean types. But they concluded that the sustained accumulation and exudation of citrate is mainly responsible for the genotypic differences in Al tolerance. In the present work, 48 h after Al exposure the malate transporter regulator protein was highly expressed in contrast with the observation of Silva et al. [29]. We postulate that* Cys2His2* might regulate the expression of other Al tolerance genes in addition to malate transporter. It is also possible that malate biosynthesis becomes a limiting step or malate might indeed play a major role in soybean Al-tolerance contrary to earlier conclusions. *ADR6* transcription factors were previously reported as Al tolerance genes [14, 18]. In the present study, *ADR6* was highly upregulated (14-fold, Table 2). The plant hormone auxin and *ADR6* exhibit opposite behavior in plant roots under Al stress. Al has been shown to inhibit auxin biosynthesis and transport genes as one possible mechanism of its toxicity [18]. On the contrary, *ADR6*—an auxin downregulated transcription factor is induced under Al stress perhaps mimicking auxin's role of promoting root growth. These observations suggest that *Cys2His2* and *ADR6* transcription factors are important modulators of soybean molecular response to Al stress.

#### 3.5.2. Genes Related to Transporters

Transporters, specifically malate (*ALMTs*) and citrate (*MATE*) transporters are the first Al tolerance genes cloned in plants and represent the well-characterized Al tolerance mechanism in a wide range of plant species [5, 8]. None of the family members of these two genes were detected in the present study which could be due to constitutive expression. In contrast, an *ABC* transporter, a multidrug resistance glutathione-S-transferase-exporting ATPase (Gma.14080, Table 2), was upregulated 27-fold at 48 h post treatment in the present study, which could detoxify xenobiotics by transporting glutathione-S-transferase conjugated toxin to the vacuole from sensitive sites in symplast. The involvement of *ABC* transporters in Al tolerance mechanism is widely documented [15, 18, 30, 31]. Other Al-induced transporters included heavy metal ion transport proteins (Gma.17184 and Gma.24625), lipid transport proteins (DQ222982 and Gma.17184), carbohydrate transport protein (Gma.11888), and coatomer protein complex subunit 2-protien—a polypeptide complex for membrane trafficking (Gma.1654) (Table 2). Heavy metal transport proteins are either located in plasma membrane or subcellular membranes and detoxify heavy metals by exporting metal-ligand complexes out of the cell or by sequestration or compartmentalization of the complex in the vacuole. The internal detoxification mechanism of Al involves formation of Al-organic acid complexes and subsequent transport of the complex by transport proteins to leaf vacuoles in Al hyperaccumulating plants that are adapted to acid soils [1, 9, 10, 32]. Similar mechanism might operate in cultivated plants, and the heavy metal binding proteins upregulated here might function in such pathway.

Lipid and sugar transport proteins are among other transporters detected. Lipid transport proteins transport lipids to cell wall for biosynthesis of cutin layers and surface waxes as a defense mechanism against pathogen attack [33]. They are also induced by abiotic stresses including aluminum [15, 33]. Lipid transport proteins loosen cell wall in a nonhydrolytic mode and enhance cell elongation, a role traditionally attributed to expansins [34]. Aluminum stress inhibits root growth by restricting cell wall extension [1]; hence, there should be a significance to the upregulation of lipid transport proteins under Al stress. Plant sugar transporters have been reported to be induced by pathogen attack and Al stress [18, 35], as is the case in the present study (Gma.11888, Table 2).

#### 3.5.3. Genes Related to Stress Response

Aluminum toxicity has been shown to elicit a wide range of stress-related proteins [19, 20, 36, 37]. In this study, genes known to be responsive to pathogens, oxidative stress, toxins, or Al were classified under this category. Several pathogenesis-related proteins including syringolide-induced protein, acidic endochitinase, PR-5, basic secretory protein, pathogenesis related protein STH-2, and proteinase inhibitors were upregulated at 48 h post Al treatment (Table 2). The confluence between plant molecular response to aluminum toxicity and pathogen infection likely arises from the fact that both cause oxidative stress. However, the role of pathogenesis-related proteins in Al tolerance is equivocal. Overexpression of peroxidase and proteinase inhibitor genes in Arabidopsis did not improve Al tolerance for the transformed plants relative to controls [38]. On the other hand, overexpressing pepper basic pathogenesis-related protein 1 gene in tobacco resulted in enhanced tolerance to heavy metal cadmium and pathogen infection [39]. 

Other Al-upregulated stress-related genes included carbohydrate oxidase, glutathione-S-transferase, and glutathione-based reductase (Tables 1 and 2). Carbohydrate oxidase and cell wall peroxidases have been reported to provide protection against pathogens by generating hydrogen peroxide from carbohydrate substrates in the apoplast [14]. Hydrogen peroxide has antimicrobial property and also acts as signal molecule for defense genes expression. In the case of aluminum, the activity of these enzymes is correlated with plant Al sensitivity [40, 41]. Glutathione-S-transferase and glutathione-based reductase are the key enzymes of cellular detoxification and antioxidation system [42]. Glutathione reductase catalyses the conversion of oxidized glutathione to reduced form. Glutathione-S-transferase conjugates toxins and electrophilic compounds to reduced glutathione. The glutathione-conjugated toxin is then exported out of the cell or into the vacuole by the *ABC* transporter proteins discussed above. The concurrent upregulation of glutathione-based reductase, glutathione-S-transferase, and *ABC *transporter protein suggests that PI 416937 soybean may guard itself against Al by extruding Al out of the cell or by compartmentalization of Al to the vacuole. Yet there are conflicting evidences with respect to the role of the glutathione defense system in plant Al tolerance. Overexpression of glutathione-S-transferase in *Arabidopsis thaliana* has been shown to enhance plant Al tolerance [38]. On the other hand, Maron et al. [17] found more oxidative stress genes upregulation in Al sensitive cultivar of maize than in Al tolerant cultivar and argue that oxidative stress genes upregulation is a symptom of Al toxicity rather than a tolerance mechanism, an assertion that is supported by findings of [43]. In addition, these genes are responsive to several biotic and abiotic stress factors and, therefore, should not be regarded as major Al tolerance genes while partial role is certainly possible.

#### 3.5.4. Genes Related to Cellular Metabolism

Genes involved in catabolic or biosynthesis of various metabolites were differentially expressed. The most interesting ones from Al tolerance perspective are genes for biosynthesis of ascorbic acid and genes encoding cytochrome P450 and endo-xyloglucan transferases/hydrolases. All were upregulated in the present study, and the last two were previously reported to be upregulated in Arabidopsis [19, 20] and wheat [15, 16] roots under Al stress. Ascorbic acid is an important component of cellular antioxidation system. Oxidative stress is one aspect of Al toxicity, and maintenance of cellular ascrobate homeostasis has been reported to be an essential component of plant Al tolerance [16]. Cytochrome P450 may serve as monooxygenase in the biosynthetic pathways for lignin, defense compounds, hormones, pigments, fatty acids, and signaling molecules or in the detoxification pathway to catalyze the breakdown of numerous endogenous and exogenous toxic compounds [44]. We detected two genes (Gma.28852 upregulated 43-fold and Gma.29655- upregulated 15-fold) which code for cytochrome P450 (Table 2). Gma.28852 encodes protein involved in pathways of ascorbate metabolism, coumarine and phenylpropanoid biosynthesis, and gamma hexachlorohexane degradation. Endoxyloglucan hydrolases are cell wall metabolism enzymes. Members of this family of enzymes have been implicated in Al tolerance [16, 18–20]. There is a causal relationship among endoxyloglucan hydrolases, cell wall composition, and Al tolerance. Al induced increases in cell wall pectin and hemicellulose increases plant Al sensitivity [43]. Pectin and hemicellulose form complexes with Al resulting in increased cell wall rigidity and reduced cell extension and growth [27, 43, 45]. Endoxyloglucan hydrolases appear to relax the Al-rigidified cell wall presumably by hydrolyzing the Al-sugar complexes.

#### 3.5.5. Genes Related to Cell Signaling

Perception of stress signal by the cell is the starting point for cascade of events leading to gene expression and change in cell metabolism in response to a stress factor. Aluminum perception and signaling is currently poorly understood. Cell wall-associated receptor kinase (WAK1) was the first Al signaling gene discovered [46], but there is no evidence that demonstrate, WAK1's major role in Al tolerance [1]. Microarray analyses have shown kinases, phosphates, and EF hand Ca^2+^ binding proteins as possible components of Al signaling pathway [16, 18]. In the present work, a Ca^2+^ sensor protein (Gma.35830), calcium-binding EF hand family protein (Gma.7726), oxidative signal kinase (Gma.8262), and a gene for growth factor phytosulfokines precursor (BK0001191) were upregulated 48 h post Al treatment (Table 2). The phytosulfokines growth factor is a novel Al-induced gene, and it is involved in cell proliferation and growth, characteristics that confer Al tolerance.

## 4. Conclusion

We conducted a transcriptome analysis in Al-tolerant soybean line PI 416937 to identify potential genetic factors underlying Al tolerance trait. Our results uncovered several genes which might potentially have influence on soybean Al tolerance. Among these, two transcription factors, cell wall metabolism enzymes and a cell proliferation gene are particularly interesting from perspective of the physiological and molecular mechanisms of plant Al tolerance. The first transcription factor, *Cys2His2* zinc finger protein, coregulates molecular response to proton and aluminum toxicities, the major acid soil stress factors [28]. The second transcription activator, *ADR6 *is an auxin downregulated gene. Al suppresses auxin biosynthesis and transport in root system which might be one possible mechanism of Al induced root growth inhibition [18]. Conversely, *ADR6* is triggered under Al stress probably acting in a parallel pathway to auxin to restore root growth under Al stress. Root cell wall rigidification by Al binding is one principal mechanism of Al toxicity. Cell wall metabolism enzymes and proteins are induced under Al stress and may counteract Al effects on root cell walls. It is increasingly evident that these proteins as well as cell wall pectin and hemicellulose content are important determinants of Al tolerance in cereals [3, 4, 43]. Evidence from this study also implies that cell wall remodeling enzymes and proteins may play role in soybean Al tolerance. Inhibition of cell division and proliferation is another major mechanism of Al toxicity. We identified a novel cell proliferation stimulating gene phytosulfokines growth factor which might reverse this effect of Al. Taken together; our findings provide important insights into the molecular mechanisms of aluminum tolerance in soybean. The genes we identified may guide efforts to improve plant Al tolerance trait.

## Figures and Tables

**Table 1:** Aluminum-regulated genes in soybean genotype PI 416937 2 h post aluminum treatment^†^.

Unigene ID	Fold change	Average ± SD	Functional category	Annotation	*e*-value
Gma.18664	32.55 (down)	−0.88 ± 0.06	Stress response	Anionic peroxidase/ oxidative stress	6*e* − 66
Gma.4152	29.89 (up)	4.39 ± 0.13	Stress response	Trypsin and protease inhibitor	2*e* − 20
Gma.17961	29.13 (up)	4.63 ± 0.14	Stress response	Soybean oleosin isoform B	2*e* − 22
Gma.26984	27.751 (up)	3.96 ± 0.05	Stress response	Putative protease inhibitor	4*e* − 35
Gma15007	24.75 (up)	3.77 ± 0.24	Metabolism	Ferredoxin 2 protein	2*e* − 46
Gma.29855	11.98 (up)	1.81 ± 0.15	Metabolism	Ribulose-1, 5-bisphosphate carboxylase	5*e* − 100
Gma.18110	11.36 (up)	2.87 ± 0.19	Unclassified	Cp12-2 Protein/peptide cross-linking	9*e* − 29
Gma.26937	8.29 (down)	−0.61 ± 0.03	Unclassified	Unknown	—
Gma.10987	8.03 (up)	2.76 ± 0.09	Metabolism	Ribulose bisphosphate carboxylase	1*e* − 33
BE024005	7.58 (up)	3.41 ± 0.27	Stress response	Glutaredoxin family protein- /glutathione-dependent reductase	6*e* − 24
Gma.2577	7.3 (down)	−0.86 ± 0.02	Metabolism	Hydrolase family protein	7*e* − 30
Gma.21354	6.51 (up)	2.59 ± 0.12	Transcription factor	NAc1 domain protein Plant development protein	1*e* − 61
Gma.4156	6.41 (up)	1.26 ± 0.24	Transcription factor	Zinc finger protein/transcription factor (CCCH-type family)	4*e* − 10
Gma.32658	5.78 (up)	2.25 ± 0.31	Unclassified	Hypothetical protein	1*e* − 44
Gma.12121	5.36 (up)	2.23 ± 0.07	Unclassified	Hypothetical protein	9*e* − 13
Gma.6487	4.62 (up)	2.33 ± 0.19	Unclassified	Unknown	—
Gma.15538	4.47 (up)	2.16 ± 0.21	Stress response	Glutaredoxin family protein (arsenate reductase)	7*e* − 39
Gma.28376	4.43 (down)	−0.02 ± 0.28	Stress response	Syringolide-induced protein B13-1-9 hypersensitive response	6*e* − 102
Gma.12481	4.39 (up)	2.52 ± 0.19	Unclassified	Hypothetical protein	2*e* − 44
Gma.4226	4.36 (up)	1.53 ± 0.16	Stress response	ATPP2-A13 protein/wound response	2*e* − 44
Gma.1248	4.09 (up)	2.50 ± 0.16	Signaling	nod33 protein (putative phosphatase)	6*e* − 88
BQ629821	3.97 (up)	2.01 ± 0.17	Transcription factor	My family transcription factor	5*e* − 14
Gma.1043	3.86 (up)	1.03 ± 0.07	Unclassified	Hypothetical protein	6*e* − 21
Gma.31382	3.79 (up)	2.04 ± 0.07	Transcription factor	Bzip transcription factor (bzip 105)	0.0
BQ785779	3.75 (up)	1.68 ± 0.13	Unclassified	Unknown	—
Gma.4710	3.54 (up)	1.63 ± 0.21	Unclassified	Hypothetical protein	3*e* − 19
Gma.27015	3.53 (up)	1.67 ± 0.08	Unclassified	Octicosapeptide PB1 domain protein	2*e* − 34
Gma.23849	3.51 (up)	1.01 ± 0.05	Unclassified	Unknown	—
Gma.4216	3.39 (up)	3.01 ± 0.01	Metabolism	Endo-xyloglucan transferase/hydrolase	3*e* − 64
AW733463	3.38 (up)	1.86 ± 0.11	Unclassified	Unknown	—
BF595565	3.35 (up)	1.50 ± 0.06	Metabolism	SDP1 (sugar dependent 1)/ triacylglycerol lipase	1*e* − 66
CF807342	3.29 (up)	2.45 ± 0.20	Unclassified	Hypothetical protein	2*e* − 18
Gma.32376	3.27 (up)	2.38 ± 0.02	Transcription factor	BLH1 (embryo sac develop arrest 29)	4*e* − 21
Gma.27837	3.16 (up)	1.66 ± 0.05	Unclassified	Hypothetical protein	7*e* − 29
Gma.4149	3.13 (down)	0.42 ± 0.04	Unclassified	Unknown	—
Gma.19917	3.12 (up)	1.48 ± 0.02	Metabolism	CTP synthase/biosynthesis	3*e* − 54
Gma.34551	3.08 (up)	1.71 ± 0.04	Signaling	MARD1 (mediator of ABA-regulated Dormancy1)	2*e* − 20
Gma.3429	3.02 (up)	2.01 ± 0.10	Metabolism	2-oxoisovalerate dehydrogenase	3*e* − 54
Gma.32595^††^	5.25 (up)	1.32 ± 0.45	Stress response	Glutathione s-transferase	7*e* − 111
Gma.20326^†††^	3.2 (down)	−1.44 ± 0.20	Unknown		—

^†^Significance threshold (*P* < .01, Fold change >= 3);^ ††^from the 12 h post treatment, ^†††^ from 72 h post treatment; up: upregulated; down: downregulated; *e*-value: the probability that the match between the gene and its annotation has no biological basis. Fold change: absolute value of the ratio of gene expression under Al to gene expression of untreated control. SD: standard deviation.

**Table 2:** Aluminum regulated genes in soybean genotype PI 416937 48 h post treatment^†^.

Unigene ID	Fold change	Average ± SD	Functional category	Annotation	*e*-value
Gma.6089	226.57 (up)	6.48 ± 1.80	Unclassified	Unknown	—
BM139770	176.22 (up)	5.91 ± 0.80	Unclassified	Unknown	—
Gma.2586	154.32 (up)	5.86 ± 0.94	Unclassified	Unknown	—
Gma.1654	130.42 (up)	4.69 ± 0.64	Transport	Coatomer protein complex subunit 2 protein transporter	2*e* − 51
Gma.26937	115.43 (up)	5.43 ± 1.37	Unclassified	Unknown	—
Gma.35222	113.94 (up)	4.67 ± 0.56	Stress response	Syringolide-induced protein B13-1-9 defense protein	4*e* − 64
Gma.24062	89.44 (up)	5.15 ± 1.37	Unclassified	Unknown	—
Gma.8048	79.07 (up)	5.03 ± 0.94	Unclassified	Unknown	—
Gma.27466	72.87 (up)	4.96 ± 1.57	Unclassified	Unknown	—
Gma.12326	71.14 (up)	4.84 ± 1.90	Unclassified	Unknown	—
Gma.6948	69.75 (up)	4.69 ± 1.27	Unclassified	Unknown	—
BU551397	65.63 (up)	4.37 ± 0.70	Unclassified	Hypothetical protein	2*e* − 15
Gma.2523	61.59 (up)	4.78 ± 0.96	Stress response	Secretory protein (R14 protein soybean-defense protein)	6*e* − 64
Gma.35601	59.51 (up)	3.61 ± 0.43	Transport	Heavy-metal transport/detoxification	2*e* − 19
Gma.6948	58.09 (up)	4.45 ± 0.98	Unclassified	Unknown	—
Gma.16246	55.90 (up)	4.27 ± 0.59	Unclassified	BAP2 (BON associated protein 2)	1*e* − 15
Gma.30731	54.43 (up)	4.14 ± 0.90	Unclassified	Unknown	—
BI967874	53.43 (up)	4.4 ± 1.35	Unclassified	Unknown	—
Gma.4526	50.59 (up)	4.38 ± 1.02	Transcription factor	Zinc finger (C2H2 family protein)	7*e* − 27
Gma.25191	47.41 (up)	5.06 ± 1.11	Unclassified	Unknown	—
Gma.9397	47.24 (up)	4.28 ± 0.98	Stress response	Syringolide-induced protein B13-1-9/defense protein	1*e* − 53
Gma.25462	46.34 (up)	4.34 ± 0.65	Transcription factor	WRKY19 DNA-binding protein 19	1*e* − 62
BU579058	45.35 (up)	4.44 ± 1.44	Metabolism	N-acetyltransferase activity	2*e* − 40
Gma.28852	43.12 (up)	4.10 ± 0.58	Metabolism	Cytochrome P450	0.0
Gma.27514	41.91 (up)	3.91 ± 0.98	Stress response	Basic secretory protein/defense protein	3*e* − 69
Gma.23347	41.67 (up)	4.08 ± 1.79	Unclassified	Unknown	—
Gma.22079	41.46 (up)	4.34 ± 0.70	Stress response	Glutathione s-transferase	5*e* − 57
Gma.32994	40.77 (up)	3.71 ± 0.62	Stress response	Acidic endochitinase (chitinase III-A)	7*e* − 93
Gma.27743	39.93 (up)	4.14 ± 0.98	Unclassified	Unknown	—
Gma.1622	39.56 (up)	3.93 ± 1.02	Unclassified	Hypothetical protein/ABC transporter like	8*e* − 31
Gma.36756	36.68 (up)	4.27 ± 1.08	Transcription factor	WRKY17 protein/transcription factor	1*e* − 101
Gma.5622	32.77 (up)	4.29 ± 0.75	Unclassified	Unknown	—
Gma.26204	32.06 (up)	3.82 ± 0.46	Metabolism	Transferase/transferase activity	2*e* − 29
Gma.27239	31.70 (up)	3.83 ± 1.35	Unclassified	Unknown	—
Gma.36287	31.08 (up)	4.56 ± 0.48	Metabolism	Carboxylesterase/lipase activity	3*e* − 64
Gma.28246	30.45 (up)	3.54 ± 0.64	Unclassified	Unknown	—
Gma.36753	30.41 (up)	4.50 ± 0.74	Transcription factor	WRKY 30(DNA binding protein	1*e* − 104
Gma.8565	29.01 (up)	3.45 ± 0.70	Metabolism	Hydrolase /xyloglucan endotransglycosylase	5*e* − 18
Gma.7861	28.48 (up)	3.48 ± 1.34	Unclassified	Unknown	5*e* − 20
Gma.32790	28.00 (up)	3.63 ± 0.99	Stress response	Band 7 family protein/hypersensitive inducible reaction protein 1	3*e* − 27
Gma.7697	27.48 (down)	−3.66 ± 0.81	Unclassified	Hypothetical protein/31 kDa glycoprotein	2*e* − 137
Gma.31827	27.44 (up)	4.22 ± 0.65	Transcription factor	WRKY70/DNA-binding protein 70	5*e* − 87
Gma.21022	27.36 (up)	4.13 ± 1.18	Unclassified	Unknown	—
Gma.28273	27.29 (up)	3.84 ± 0.87	Transcription factor	NAC6 /NAC domain protein/apical elongation plant development protein	2*e* − 156
Gma.35830	27.20 (up)	3.48 ± 0.77	Signaling	Regulation of gene silencing/calcium-sensor	9*e* < ?*b* *h* *l* *t*?>−<?ehlt?>33
Gma.8262	26.92 (up)	3.73 ± 1.33	Stress response	AGc 2-1(oxidative signal-inducible kinase	3*e* − 57
Gma.14080	26.50 (up)	3.81 ± 0.80	Transport	Similar to ATMRP3/multidrug resistance glutathione s-conjugate-exporting ATPase	8*e* − 11
Gma.27371	25.99 (up)	3.78 ± 1.33	Unclassified	Unknown	—
BM523736	25.34 (up)	3.76 ± 0.83	Metabolism	Transferase family protein	1*e* − 23
Gma.28330	25.32 (up)	3.84 ± 1.53	Unclassified	Calcium-binding protein	7*e* − 23
Gma.34717	24.87 (up)	3.83 ± 1.15	Unclassified	Unknown	—
Gma.26682	24.58 (up)	3.95 ± 1.15	Unclassified	Unknown	—
Gma.17184	24.42 (up)	3.77 ± 0.52	Transport	Glycolipid-binding/transport protein	2*e* − 57
Gma.11888	24.39 (up)	3.19 ± 0.25	Transport	ATPP2-B10 (pheloem protien2) carbohydrate binding	6*e* − 27
Gma.4222	23.57 (up)	3.14 ± 0.97	Unclassified	Hypothetical protein	2*e* − 57
Gma.26712	23.39 (up)	2.82 ± 0.18	Unclassified	Unknown	—
Gma.33327	23.16 (up)	3.56 ± 0.29	Transcription factor	Transcription factor	2*e* − 166
Gma.17019	23.08 (up)	3.67 ± 1.54	Unclassified	Unknown	—
Gma.33178	23.07 (up)	3.81 ± 1.54	Unclassified	Plastocyanin-like domain-containing copper ion binding	3*e* − 33
Gma 35364	22.81 (up)	3.55 ± 0.40	Stress response	FAD-linked oxidoreductase 1/carbohydrate-oxidase	1*e* − 52
Gma.15839	22.63 (down)	−4.00 ± 0.36	Metabolism	GDSL-motif lipase/hydrolase	2*e* − 39
Gma.6948	22.54 (up)	3.73 ± 1.24	Unclassified	Unknown	—
Gma.2821	22.36 (up)	3.40 ± 0.95	Stress response	PR-5 protein (pathogenesis related)	4*e* − 134
Gma.8628	21.20 (up)	3.19 ± 1.11	Unclassified	Unknown protein	—
BQ473604	20.51 (up)	3.43 ± 0.83	Unclassified	Hypothetical protein	8*e* − 46
Gma.9913	20.34 (up)	3.81 ± 0.44	Unclassified	Unknown protein	—
Gma.144	20.01 (up)	3.72 ± 1.00	Transport	Nodulin protein/transport function	0.0
Gma.34099	19.67 (up)	2.93 ± 0.93	Unclassified	Hypothetical protein	2*e* − 77
Gma.4305	19.63 (up)	3.82 ± 0.62	Stress response	Glutathione s-transferase (GST 15)	7*e* − 128
Gma.4336	19.56 (up)	3.16 ± 0.98	Unclassified	Unknown	—
Gma.24625	19.40 (up)	3.50 ± 1.12	Transport	Heavy metal transport/detoxification	2*e* − 20
Gma.7726	19.18 (up)	3.36 ± 1.22	Signalling	Calcium-binding EF hand family protein	4*e* − 25
Gma.26531	18.95 (up)	3.55 ± 1.31	Transcription factor	Zinc finger (C3HC4-type ring familyn)	2*e* − 26
Gma.34717	18.94 (up)	3.46 ± 1.24	Unclassified	Unknown	—
BE822282	18.64 (up)	2.87 ± 0.66	Unclassified	Unknown	—
Gma.27062	18.22 (up)	2.96 ± 0.95	Transcription factor	NAc domain containing protein 2 plant development/apical elongation	3*e* − 103
Gma.4478	18.03 (up)	3.54 ± 1.03	Unclassified	Hypothetical protein	2*e* − 10
Gma.24807	18.01 (up)	3.09 ± 0.76	Unclassified	Unknown	—
BK000119.1	17.98 (up)	3.36 ± 0.87	Cell cycle	Phytosulfokines 4 precursor/ growth factor cell differentiation, cell proliferation	3*e* − 19
Gma.29479	17.92 (up)	3.16 ± 0.52	Unclassified	Unknown	—
Gma.10956	17.76 (up)	3.32 ± 0.74	Stress response	Similar to pathogenesis-related protein (STH-2)	1*e* − 40
BI967589	17.25 (up)	3.08 ± 0.49	Unclassified	Unknown	—
Gma.17184	17.16 (up)	2.94 ± 0.11	Transport	Heavy-metal-associated domain containing protein metal ion transport	1*e* − 10
Gma.24561	17.08 (up)	3.15 ± 0.31	Unclassified	Unknown	—
Gma.21739	17.05 (up)	3.33 ± 0.82	Metabolism	AAA-type ATpase protein/ATpase activity	5*e* − 39
Gma.17184	16.89 (up)	3.27 ± 0.63	Transport	Glycolipid-binding protein/glycolipid transport	
Gma.4366	16.62 (up)	3.29 ± 1.27	Metabolism	VTc2 (Vitamin C defective 2)/ L-ascorobic-acid biosynthesis	1*e* − 36
Gma.26405	15.37 (up)	3.04 ± 0.83	Unclassified	Unknown	—
DQ222982	15.09 (up)	3.42 ± 1.04	Transport	Lipocalin/ fatty acid transport	2*e* − 107
Gma.17929	15.05 (up)	3.12 ± 0.58	Metabolism	Transferase family protein	8*e* − 76
Gma.21512	14.99 (up)	2.71 ± 0.78	Unclassified	Unknown	—
BE440732	14.85 (up)	3.48 ± 0.97	Unclassified	Unknown	—
Gma.29663	14.64 (up)	2.52 ± 1.04	Unclassified	Unknown	—
Gma.31861	14.60 (up)	3.09 ± 1.07	Unclassified	Unknown	—
Gma.29655	14.52 (up)	3.00 ± 0.34	Metabolism	CytochromeP50 subfamily B polypeptide 1	3*e* − 58
Gma.11257	14.51 (up)	2.65 ± 0.86	Unclassified	Hypothetical protein exo-1, 3-beta-glucanase precursor	9*e* − 64
Gma.26640	14.48 (up)	2.93 ± 1.18	Unclassified	Unknown	—
Gma.28243	14.18 (up)	3.28 ± 0.85	Unclassified	Unknown	—
CD394418	14.12 (up)	3.08 ± 1.24	Metabolism	Ribulose-1, 5-bisphosphate carboxylase	2*e* − 30
Gma.1527	14.09 (down)	−2.26 ± 1.40	Metabolism	Dihydroflavonol reductase (anthocyanin biosynthesis)	0.0
Gma.25234	14.08 (up)	2.96 ± 0.18	Transcription factor	WRKY43 protein	4*e* − 131
Gma.28057	13.62 (up)	2.52 ± 0.68	Transcription factor	Sali5-4a protein (ADR6)	8*e* − 60
Gma.8480	13.58 (up)	3.20 ± 0.69	Stress response	Resistance protein LM12	0.0
Gma.28756	13.53 (up)	2.89 ± 0.53	Unclassified	Unknown	—
BM177218	13.45 (up)	3.14 ± 0.45	Unclassified	Unknown	—
BG551078	13.37 (up)	3.09 ± 0.42	Unclassified	Conserved hypothetical protein	6*e* − 11
Gma.728	13.28 (up)	2.78 ± 0.99	Unclassified	Unknown	—
Gma.35332	13.23 (up)	3.45 ± 0.73	Unclassified	Unknown	—
Gma.26712	13.17 (up)	3.09 ± 1.01	Unclassified	Unknown	—

^†^Significance threshold (*P* < .01, Fold change >= 3); up: upregulated, down: downregulated; *e*-value: the probability that the match between the gene and its annotation has no biological basis. Fold change: absolute value of the ratio of gene expression under Al to gene expression of untreated control. SD: standard deviation.
